# Supplementary material for: Pain Improvement in Parkinson’s Disease Patients Treated with Safinamide: Results from the SAFINONMOTOR Study
Source: J Pers Med. 2021 Aug 16;11(8):798. doi: 10.3390/jpm11080798 (PMC8402181; doi:10.3390/jpm11080798)
Supplement: Supplementary file 1 [file jpm-11-00798-s001.zip › Table S1. SAFINOMOTOR.PAIN.pdf]

Table S1. Data about sociodemographic aspects, comorbidities, antiparkinsonian drugs and other therapies at baseline.

	N			N	
Age	50	68.50 ± 9.12	Time from diagnosis of PD	48	6.39 ± 5.06
Gender (females) (%)	50	58			
Race (%)	50		Motor fluctuations (%)	50	78
- Caucasian	50	98	Dyskinesia (%)	50	30
- Indian	50	2	Cognitive impairment (%)	50	4
Civil status (%):	27		Treatment for PD (%):	50	
- Married		70.4	- Levodopa		94
- Widowed		22.2	- Dopamine agonists:		66
- Other		7.4	* Pramipexole		36
			* Ropinirole		14
Living style (%)	29		* Rotigotine		18
- With the partner		65.5	- COMT inhibitor		34
- With another family member		27.6	*Entacapone		16
- Other		7.2	*Opicapone		18
			- Amantadine		8
Habitat (%):	30		- Anticholinergic drug		0
- Rural (< 5.000)		13.4	L-dopa daily dose (mg)		612.23 ± 423.4
- Semiurban (5.000-20.000)		23.3	DA daily dose (mg)		234.33 ± 96.19
- Urban (> 20.000)		63.3	LEDD (mg)		810.21 ± 518.14
Comotbidities (%):	50		Other treatments (%):	50	
- Arterial hypertension		40	- Antidepressant		32
- Diabetes mellitus		20	- Benzodiazepine		38
- Dyslipemia		36	- Antipsychotic		4
- Cardiopathy		10	- Analgesic		22
- Cardiac arrhythmia		12	NSAIDs		10
- Smoking		10	Opioid		10
- Alcohol consumption		12	Paracetamol		8
			AEDs		8
			Number of anti-PD drugs	50	2.27 ± 0.94
			Total number of drugs	50	6.36 ± 3.14

The results represent % or mean ± SD. Cognitive impairment is with regards to the opinion of the neurologist (no dementia; MMS ≥ 26).

AEDs, antiepileptic drugs; COMT, catechol-O-methyltransferase; DA, dopamine agonist; LEDD, levodopa equivalent daily dose; NSAIDs, non-steroidal anti-inflammatory drugs; PD, Parkinson´s disease.
