# Supplementary material for: Pain Improvement in Parkinson’s Disease Patients Treated with Safinamide: Results from the SAFINONMOTOR Study
Source: J Pers Med. 2021 Aug 16;11(8):798. doi: 10.3390/jpm11080798 (PMC8402181; doi:10.3390/jpm11080798)
Supplement: Supplementary file 1 [file jpm-11-00798-s001.zip › Table S2. SAFINOMOTOR.pdf]

Table S2. Adverse events in patients from V1 to V4.

	N
Total AEs, N	21
- Nausea	3
- Dyskenia	3
- Diurnal somnolence	2
- Urinary infection	1
- Dizzness	1
- Pneumonia	1
- Respiratory insufficiency	1
- Rash	1
- Diurnal somnolence	1
- Dry mouth	1
- Insomnia	1
- Increased appetite	1
- Abdominal pain	1
- Constipation	1
- Urinary incontinence	1
- Deep brain stimulation surgery	1
Patients with at least one AE, N (%)	16 (32)
At least possibly related AEs, N	14
Patients with at least possibly* related to safinamide AEs, N (%)	9 (18)
Total SAEs, N	5
- Urinary infection	1
- Pneumonia	1
- Respiratoy insufficiency	1
- Dizzness	1
- Deep brain stimulation surgery	1
Patients with al least one SAE, N (%)	5 (10)
At least possibly* related to safinamide SAEs, N	0
Patients with at least possibly related to safinamide SAEs, N (%)	0 (0)
Patients with at least one AE leading to discontinuation, N (%)	4 (8)
Patients with at least one possibly* related to safinamide AE leading to discontinuation N (%)	1 (2)
Deaths, N (%)	0 (0)

*Considered “possibly”, “probably” or “definitely” related to treatment (safinamide). AE, adverse event; SAE, serious adverse event.
