# Supplementary material for: Pain Improvement in Parkinson’s Disease Patients Treated with Safinamide: Results from the SAFINONMOTOR Study
Source: J Pers Med. 2021 Aug 16;11(8):798. doi: 10.3390/jpm11080798 (PMC8402181; doi:10.3390/jpm11080798)
Supplement: Supplementary file 1 [file jpm-11-00798-s001.zip › Table S3. SAFINOMOTOR.pdf]

Table S3. Change in medications different of the introduction of safinamide after the baseline visit, from V1 to V4.

**Added medications or increased doses**
1 patient adding atorvastatina 1 day after V1 and **escitalopram** to 20 mg/day 93 days after V1
1 patient adding 25/100 mg/day of **carbidopa/levodopa** IR 6 days after V1
1 patient adding lansoprazol 62 days after V1 and **rotigotine** to 8 mg/day 63 days after V1
1 patient increasing the dose of **levodopa** IR from 500 to 600 mg/day 102 days after V1
1 patient adding vitamin B12 144 days after V1
1 patient adding 25/100 mg/day of **carbidopa/levodopa retard** 182 days after V1
1 patient adding rosuvastatin 182 days after V1
**Medications withdrawn or doses decreased**
1 patient withdrawing amlodipin/valsartan/hydrochlorothiazide 42 days after V1

IR, immediate release.
